# Impact of rapid maxillary expansion on palatal morphology at different dentition stages

**DOI:** 10.1007/s00784-022-04434-9

**Published:** 2022-03-10

**Authors:** Gero Stefan Michael Kinzinger, Jörg Alexander Lisson, Charlotte Buschhoff, Jan Hourfar, Heike Korbmacher-Steiner

**Affiliations:** 1 Department of Orthodontics, Saarland University, Homburg, Saar Germany; 2 Department of Orthodontics, Philipps-University Medical Center Marburg, Philipps-University, Georg-Voigt-Str. 3, 35039 Marburg, Germany

**Keywords:** Palatal morphology, Median palatine suture, Transverse palatine suture, Rapid maxillary expansion (RME)

## Abstract

**Objective:**

Rapid maxillary expansion (RME) is an established and frequently used procedure to overcome maxillary constriction. In-depth studies about morphological changes of the alveolar process and its immediate surroundings are missing. Therefore, the aim of the present study was to examine the treatment effects of a dentally anchored, rapid maxillary expander at different dentition stages upon palatal width, height and shape.

**Material and methods:**

The dental casts of 114 patients—taken immediately before and after RME—were three-dimensionally analysed. Depending on the dentition stage, the patients were divided into two groups (each *n* = 57, group 1, early mixed dentition; group 2, late mixed or permanent dentition).

**Results:**

The width increases were highly significant, both in the overall and in the individual groups (*p* < 0.001). While the width increase was greater in the posterior area than anteriorly in the early group, the widening in the late group happened significantly greater anteriorly than posteriorly. Palatal height increased anteriorly and posteriorly in both groups to a significant extent (*p* < 0.001). The height increase was more pronounced in the anterior region than in the posterior region in the late group. The palatine index according to Kim revealed a change in palatal morphology both anteriorly and posteriorly in the early group but only anteriorly in the late group.

**Conclusions:**

Maxillary expansion occurs more parallel in early treatment compared to V-shaped opening in the later treatment approach.

**Clinical relevance:**

RME is more advantageous in an early dentition.

## Introduction

The forced skeletal expansion of the maxilla, commonly known as “rapid maxillary expansion (RME)” or “rapid palatal expansion (RPE)”, was first described by the American dentist Angell [1] and remains an inherent part of orthodontic treatment measures until today. According to a survey by Korbmacher et al. [2], it is particularly used in patients showing a pronounced skeletal maxillary constriction, which is often associated with a crossbite. Grabowski et al. [3] found that patients more often have a unilateral than a bilateral crossbite and recommend orthodontic intervention as early as possible due to the progressive nature of the anomaly.

The principle of forced skeletal expansion of the maxilla is based on the application of a defined force upon skeletal structures to separate the palatine processes and the horizontal laminae of the palatine bone in order to obtain basal expansion. Depending on the age and dentition stage of the patient, a choice between four different fixed appliance variants is possible: anchored exclusively to four teeth (Hyrax type) [4], anchored to teeth and palatal mucosa (Haas type) [5], combined (hybrid) anchorage with two teeth and the jawbone [6, 7] or exclusively to the jawbone [8, 9].

The forced skeletal expansion of the maxilla affects both the median palatine suture and its surrounding sutures [10–13]. Many studies have concluded that the force peaks on the surrounding structures increase with decreasing distance from the median palatine suture. Thus, the effects are most evident on the zygomaticomaxillary and frontomaxillary sutures, while the zygomaticofrontal suture, the zygomaticotemporal suture, the nasomaxillary suture, the frontonasal suture and the internasal suture are less affected [14–18]. Greater changes are described for sutures directly connected to the maxilla than in those with an indirect connection [16, 19].

Timms [20] described the particular anatomical proximity of the paired palatine processes with the pterygoid process of the sphenoid bone. He investigated a possible age-dependent correlation between the expansion in the dentoalveolar region and the pterygoid hamuli. According to this, the palatomaxillary and pterygopalatine sutures are especially important.

Since the nasal floor and parts of the maxillary sinus floor constitute the palate, it is obvious that both structures influence each other concerning shape and dimensions: Baratieri et al. [21] described improved nose breathing through increased nasal volume after rapid maxillary expansion. Maspero et al. [22] found a positive influence upon septum deviation through an increase of longitudinal growth in the lower third of the septum, a volume increase of the nasal cavity and improved breathing through a reduction in anterior airflow resistance, and Podesser et al. [23] described an increase of the maxillary sinus, particularly in the region of the first permanent molars.

While the effects of rapid maxillary expansion on the surrounding sutures, nasal and paranasal cavities underwent an extensive scientific evaluation, there is no precise knowledge about the areas of interest directly affected by orthodontic treatment: the alveolar process and its adjacent structures of the hard palate. The present study describes for the first time the three-dimensional therapeutic effects upon these structures in a large number of patients, using plaster casts.

## Aims of the study

The data collection was used to answer the following questions:

- Are width and height of the tooth-bearing palate changes different in the anterior and posterior regions after rapid maxillary expansion depending on patient age/dentition stage?
- Are RME-induced changes measurable three-dimensionally?

## Material and methods

### *Patients (Table **1**)*

**Table 1:** Patients: Number (*n*), age, gender, average wear time of the RME, average number of Hyrax screw activations, percentage of maximum possible turns of the Hyrax screw (%), localisation of the crossbite and mandibular deviation for the total patient group and the patient groups PG 1 (early) and PG 2 (late). *M* Mean and *SD* standard deviation

Patients	Patients pooled	Patient group 1 (PG 1)	Patient group 2 (PG 2)
Number (*n*)	114	57	57
Age (years)	11.03 ± 2.59	9.13 ± 1.33	12.93 ± 2.09
Gender (m/f)	47 m/67 f	21 m/36 f	26 m/31 f
RME wear time (months)	6.15 ± 1.98	6.20 ± 2.24	6.11 ± 1.70
Number of Hyrax screw activations	24.33 ± 6.56	24.33 ± 6.41	24.33 ± 6.71
Maximum possible turns of the Hyrax screw (%)	48.67 ± 13.12	48.67 ± 12.93	48.67 ± 13.41
Crossbite (*n*) bilateral/only right/only left	61/33/20	26/17/14	35/16/6
Mandibular deviation (*n*) none/right/left	63/34/17	25/20/12	38/14/5

One hundred fourteen (67 female, 47 male) out of 167 patients who received a rapid maxillary expansion between 2010 and 2020 with an RME appliance including a Hyrax screw anchored to four teeth were included in the study, using the following inclusion criteria:

Treatment exclusively by the same orthodontist, no prior orthodontic treatment, Caucasian origin, transverse maxillary arch deficiency, uni- or bilateral crossbite, corresponding high-quality dental casts prior to treatment and immediately after RME removal and the number of Hyrax screw activations had to be almost equal in both groups. The application of these strict criteria ensured that therapeutic effects could be evaluated and interpreted without restriction.

The division into two groups was based on the dentition stage of the patient when the appliance was inserted. Patients with an early mixed dentition were assigned to group 1 (early group). Patients with a late mixed dentition, where the first premolars had to be fully erupted, and patients at the beginning of the permanent dentition were in group 2 (late group). The youngest patient was 7.16 years, and the oldest patient was 17.24 years old at treatment onset. The mean age was 11.03 ± 2.59 years (group 1, 9.13 ± 1.33 years; group 2, 12.93 ± 2.09 years). The RME appliance remained inserted for mean 6.15 ± 1.98 months (group 1, 6.20 ± 2.24 months; group 2, 6.11 ± 1.70 months). The exact data on patient age, wear time average number of Hyrax screw activations, percentage of maximum possible turns of the Hyrax screw (in %), location of the crossbite and mandibular deviation can be found in Table 1.

### Hyrax appliance

**Fig. 1:**
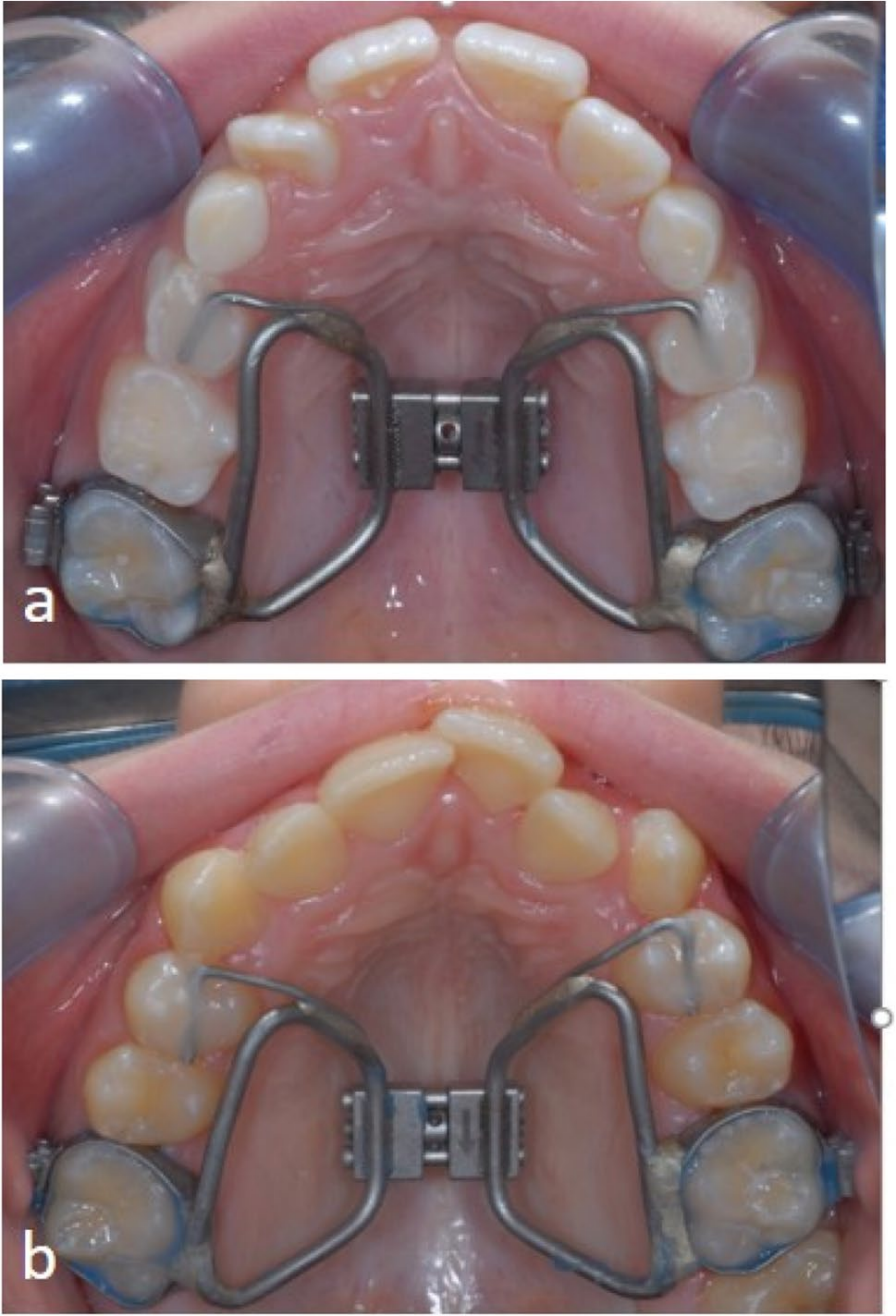
**a, b** Representative clinical pictures of the RME appliance in a patient **a** out of PG1 and **b** PG2

A Hyrax screw RME appliance with solely dental anchorage was used in all patients of this study to ensure comparability of treatment effects. This appliance (palatal screw type S with a lift height of 0.2 mm, Forestadent, Pforzheim, Germany) was fixed with two occlusal rests on the 1st premolars or deciduous molars and with two pre-fabricated bands on the first permanent maxillary molars (Fig. 1 a and b). No premolar bands were used in any of the patients; anterior fixation was gained by bonded occlusal rest on the first premolars or deciduous molars only.

The activation was performed twice daily until the therapeutically desired posterior arch width was reached. Overcorrection was planned with 25% additional widening to the correction of the maxillary deficiency.

The appliance then remained passively in situ for approximately 6 months to stabilise the treatment result (see Table 1).

### Dental casts

Two hundred twenty-eight dental casts (114 of group 1 and 114 of group 2) were measured after conversion into 3D models. The original plaster casts were made during treatment at a defined interval:

- T1: Immediately before rapid maxillary expansion.
- T2: Immediately after appliance removal.

The impressions were made using alginate from Kaniedenta (Yellow Print Alginate, Kaniedenta, Herford, Germany) and Rim-Lock impression trays. Subsequently, the impressions were cast with plaster (Kanistone Classic, hard plaster type 3, Kaniedenta, Herford, Germany) and trimmed three-dimensionally. The orthoX® scan 3D scanner (Dentaurum, Ispringen, Germany) was used to scan casts and produce their three-dimensional data set (accuracy of < 20 µm with a scan time of 45 s per model).

### Digital cast analysis

The obtained 3D data sets were virtually enhanced, trimmed and exported as an STL file through the software OnyxCeph® 3TM (Image Instruments GmbH, Chemnitz, Germany). The subsequent virtual analysis of the digital models was performed with the software 3D-Tool-Free (3D-Tool-GmbH & Co. KG, Weinheim, Germany).

**Fig. 2:**
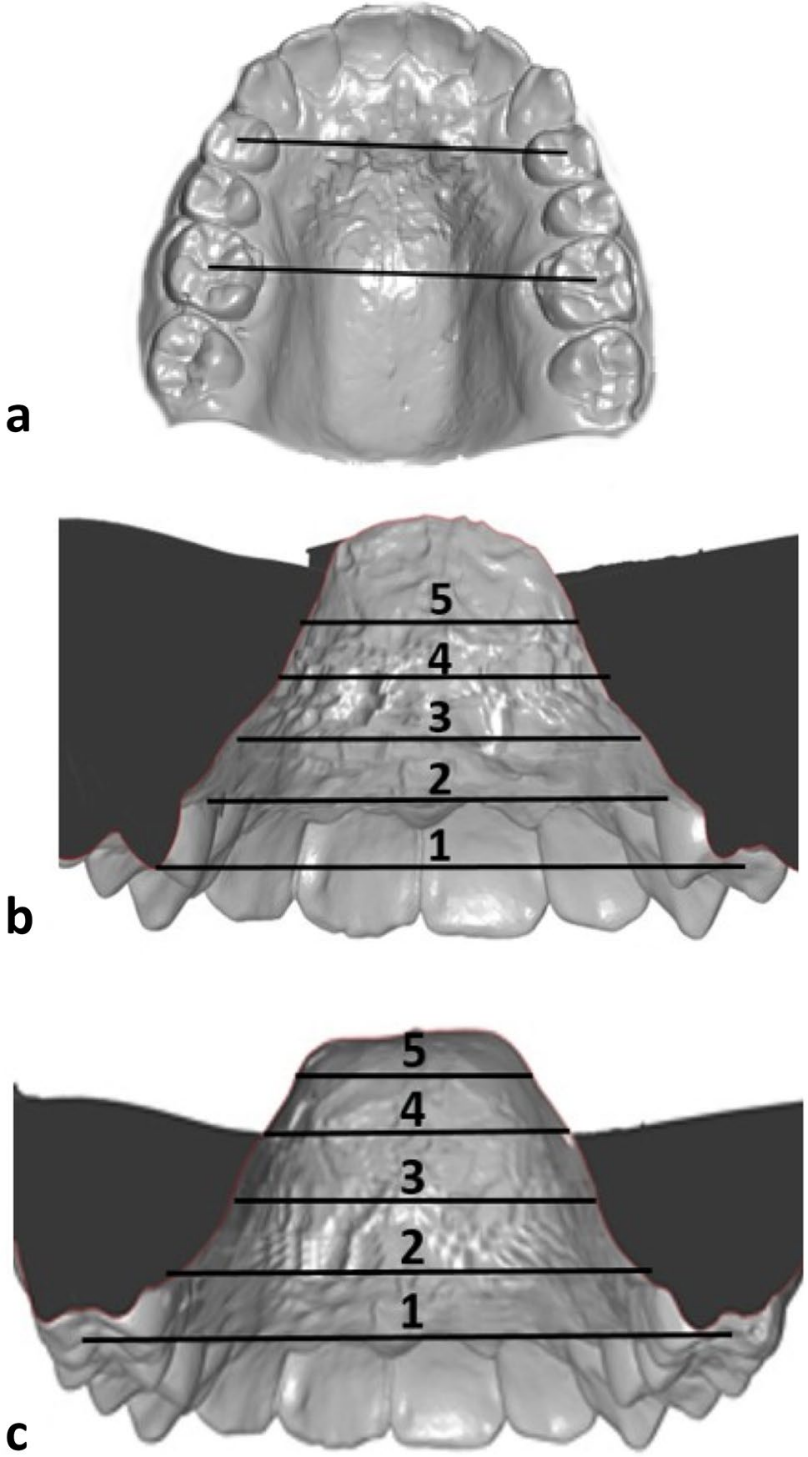
**a**–**c** Quantification of the transverse palatal dimension. **a** Analysis of dental arch width with standard reference points on the premolars or deciduous molars and on the first permanent molars. **b** and **c** Palatal width to assess the gingival-alveolar plane, measured between the most coronal points of the gingival margin at the first premolar or deciduous molars (**b**) and the first permanent molars (**c**). The width was measured in the cranial direction at different heights. The spacing of the individual measurements was always 2 mm and ended at a maximum height of 6 mm. Level 1: dental width, level 2 gingival-alveolar, level 3: level 2 + 2mm cranial movement; level 4 : level 2 + 4mm cranial movement; level 5 skeletal-basal = level 2 + 6mm cranial movement. The anterior/posterior ratio was calculated on three planes to qualify the expansion as parallel or triangular.

The dental arch width was measured anteriorly at the first premolars or deciduous molars and posteriorly at the first permanent molars (Fig. 2a). The palatine width was measured between the most coronal points of the gingival margin at the first premolars or deciduous molars and at the first permanent molars (gingival-alveolar plane). Starting from these points, the width was determined in 2 mm steps cranially up to a distance of 6 mm (skeletal-basal plane) (Fig. 2 b and c). The anterior/posterior ratio was calculated on three  planes (dental, gingival-alveolar and skeletal-basal) to qualify the expansion as parallel or triangular.

**Fig. 3:**
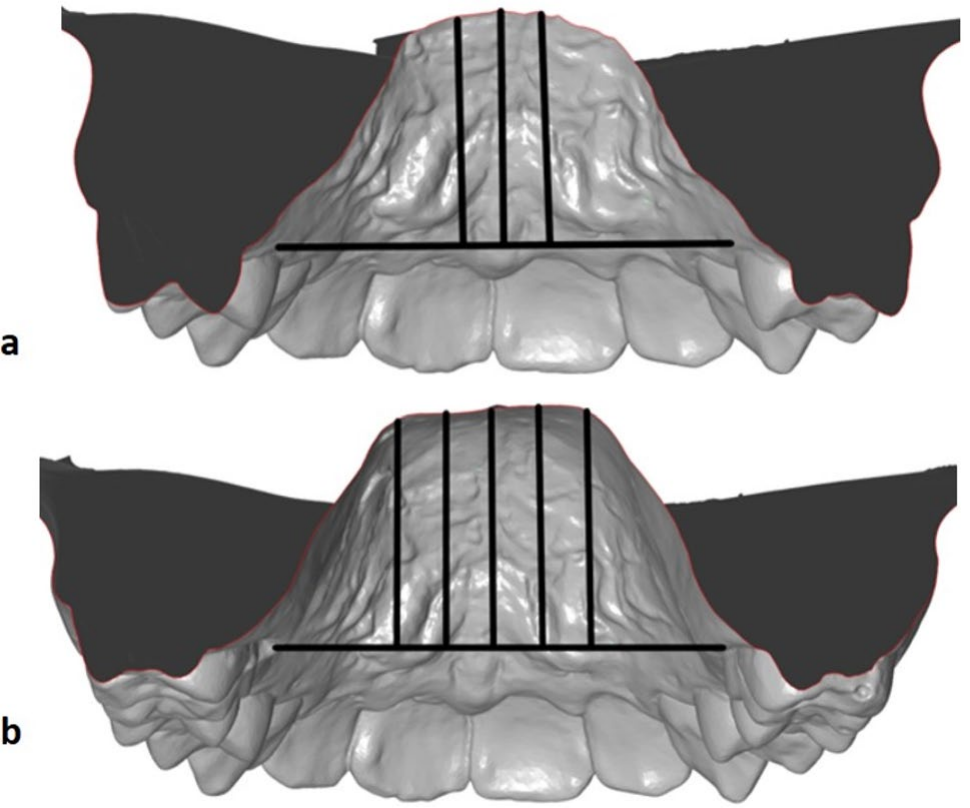
**a, b** Determination of palatal height: A point on the median raphe was connected perpendicularly to the most coronal point of the gingival margin on **a** the 1st premolars or deciduous molars and **b** the first permanent molars. For the height at the median raphe, the mean value was calculated of measurements at the reference teeth in both quadrants. **a** For the first deciduous molars or first premolars, the same measurement was taken 5 mm to the right (to the 1st quadrant) and to the left (to the 2nd quadrant) of the palatal centre, and **b** on the first permanent molars, 5 mm and 10 mm to the right and to the left of the palatal centre

For the determination of the palatine height, the raphe median line was connected perpendicularly to the most coronal point of the gingival margin on the first premolars or deciduous molars and the first permanent molars. The mean value for these measurements was calculated at the respective teeth in both quadrants. The same measurement was performed for the first deciduous molars or premolars 5 mm to the right (1st quadrant) and left (2nd quadrant) of the palatal centre and for the first permanent molars 5 mm and 10 mm to the right and left of the palatal centre (Fig. 3 a and b).

**Fig. 4:**
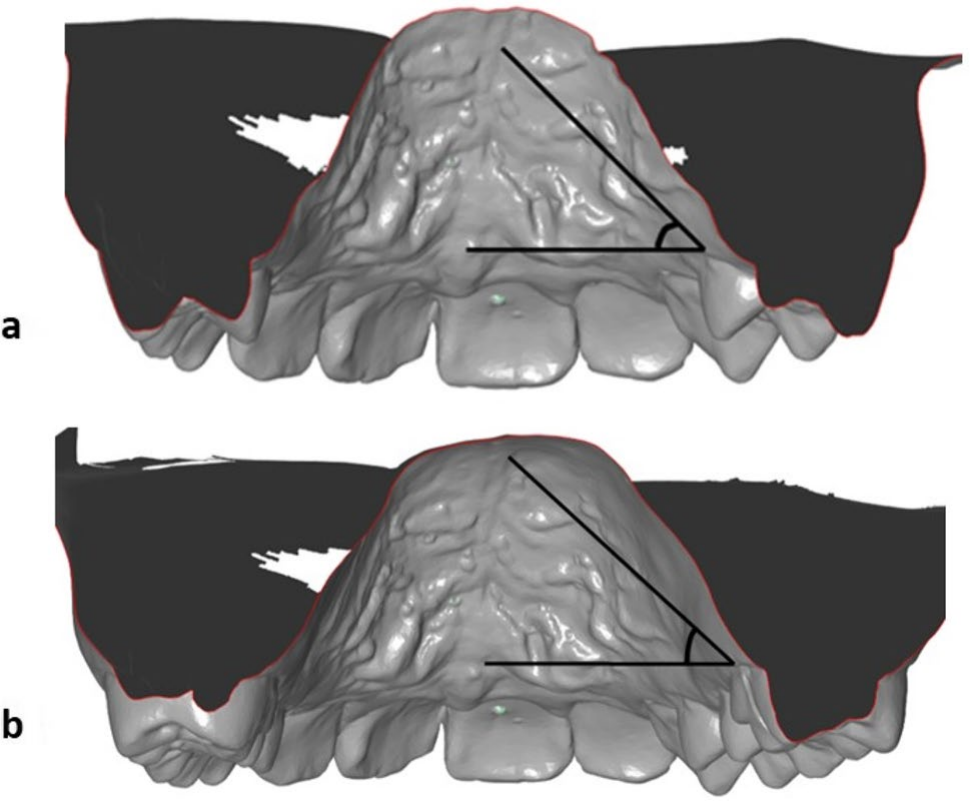
**a, b** Modified palatal index according to Kim [24] to determine the palatal shape: from the first quadrant, the angles between the horizontal reference line from the gingival margin of opposing **a** 1st premolars or deciduous molars and **b** 1st molars and the lowest point in the centre of the palatal vault were measured

The modified palatal index according to Kim et al. [24] was used to assess palatal shape changes. Starting from the first quadrant, the angles between the horizontal reference line from the gingival margin of opposing first premolars and first permanent molars and the lowest point in the centre of the palatal vault were measured (Fig. 4 a and b). The measurements were conducted using the open-source software GIMP (GNU Image Manipulation Program, The GIMP Team).

### Statistical method, error of the method

The data was collected in a structured manner using spreadsheet software (Excel®, Microsoft Corporation, Redmond, USA) on a computer with the operating system Microsoft® Windows 10 (Microsoft Corporation Redmond, USA). The collected data were subsequently imported into statistical software (SPSS® 23, Armonk, NY, USA) for Windows® (Microsoft Corporation) and analysed. Normal distribution was evaluated visually and with the Shapiro–Wilk test. Treatment-associated changes in variables were analysed using the linked *t*-test for intra-group comparisons and the independent *t*-test for inter-group comparisons. Mean and standard deviation were reported for each variable. Statistical significance was assumed at *p*-values < 0.05. The significance level was defined as follows: *p* ≥ 0.05 not significant, *p* < 0.05 significant, *p* < 0.01 highly significant and *p* < 0.001 highly significant.

To determine the combined error of the method (MF) according to Dahlberg [25], 25% of the models were randomly selected for this purpose and measured again by the same investigator after a period of 3 months. The error of the method for linear (height, width) and angular measurements was calculated with the formula MF = √(∑d2/2n) to determine the validity of the measurement method, where *d* is the difference between two measurement results and *n* is the number of duplicate measurements. The MF in the present study was < 1 for all measurements (height 0.60 mm, width 0.53 mm, angle 0.65°).

## Results

### Linear measurements

#### ***Width (Table ******2******)***

**Table 2:** Width dental, gingival-alveolar, skeletal-basal (transverse plane). Widths (in mm) in the anterior (54–64 and 14–24) and posterior (16–26) regions at five different levels of the maxilla. The dental width, the gingival/alveolar width, the width 2 mm and 4 mm cranial to the gingival/alveolar level and the skeletal/basal width are shown. *M* Mean, *SD* standard deviation, *GM* gingival margin, patient groups PG 1(early) and PG 2 (late)

	**All patients**				PG 1				PG 2				PG 1 vs. PG 2
	T1	T2	ΔT2-T1		T1	T2	ΔT2-T1		T1	T2	ΔT2-T1		
	M ± SD	M ± SD	M ± SD	*p*(intra)	M ± SD	M ± SD	M ± SD	*p*(intra)	M ± SD	M ± SD	M ± SD	*p*(intra)	*p*(inter)
54–64/14–24, dental	32.91 ± 2.02	37.23 ± 2.32	4.25 ± 1.68	< 0.001	32.20 ± 1.81	36.24 ± 1.96	3.97 ± 1.61	< 0.001	33.63 ± 1.97	38.15 ± 2.27	4.52 ± 1.71	< 0.001	0.086
54–64/14–24, GM, gingival-alveolar	24.33 ± 1.71	28.11 ± 1.76	3.78 ± 1.48	< 0.001	24.31 ± 1.58	27.88 ± 1.71	3.57 ± 1.45	< 0.001	24.35 ± 1.84	28.35 ± 1.80	4.00 ± 1.50	< 0.001	0.125
54–64/14–24, GM + 2 mm	20.02 ± 1.91	22.78 ± 2.04	2.77 ± 1.54	< 0.001	19.95 ± 1.91	22.40 ± 1.80	2.46 ± 1.36	< 0.001	20.09 ± 1.93	23.17 ± 2.21	3.08 ± 1.65	< 0.001	0.031
54–64/14–24, GM + 4 mm	15.92 ± 2.31	18.12 ± 2.36	2.21 ± 1.59	< 0.001	15.85 ± 2.08	17.72 ± 1.99	1.87 ± 1.39	< 0.001	15.98 ± 2.54	18.52 ± 2.64	2.55 ± 1.71	< 0.001	0.022
54–64/14–24, GM + 6 mm, skeletal-basal	13.10 ± 2.67	14.88 ± 2.57	1.77 ± 1.82	< 0.001	13.22 ± 2.39	14.66 ± 2.31	1.43 ± 1.79	< 0.001	12.98 ± 2.95	15.10 ± 2.81	2.11 ± 1.81	< 0.001	0.046
16–26, dental	43.36 ± 2.51	47.57 ± 2.64	4.21 ± 1.57	< 0.001	42.88 ± 2.07	47.30 ± 2.15	4.42 ± 1.47	< 0.001	43.84 ± 2.82	47.84 ± 3.05	4.00 ± 1.64	< 0.001	0.150
16–26, GM, gingival-alveolar	31.75 ± 2.45	35.23 ± 2.87	3.47 ± 1.59	< 0.001	31.59 ± 2.20	35.55 ± 2.34	3.96 ± 1.43	< 0.001	31.92 ± 2.68	34.90 ± 3.31	2.98 ± 1.61	< 0.001	0.001
16–26, GM + 2 mm	27.01 ± 2.34	29.08 ± 2.29	2.07 ± 1.58	< 0.001	26.51 ± 2.10	29.05 ± 2.13	2.54 ± 1.61	< 0.001	27.51 ± 2.47	29.11 ± 2.46	1.60 ± 1.40	< 0.001	0.001
16–26, GM + 4 mm	24.29 ± 2.56	25.70 ± 2.72	1.41 ± 1.85	< 0.001	23.38 ± 2.31	24.99 ± 2.77	1.61 ± 2.09	< 0.001	25.21 ± 2.49	26.42 ± 2.49	1.21 ± 1.58	< 0.001	0.254
16–26, GM + 6 mm, skeletal-basal	20.72 ± 3.21	21.99 ± 3.49	1.27 ± 2.05	< 0.001	19.28 ± 2.96	20.68 ± 3.44	1.40 ± 2.28	< 0.001	22.16 ± 2.80	23.30 ± 3.05	1.14 ± 1.79	< 0.001	0.512

All measured distances increase highly significant (*p* < 0.001) both in the overall group and in the two subgroups. In the late group, the increase is significantly greater anteriorly than posteriorly at all levels. In contrast to that, the patients of the early group show a greater posterior width increase in the lower three levels.

The late group patients show a significantly greater increase in anterior width than patients in the early group at all levels (*p* = 0.031, *p* = 0.022 and *p* = 0.046, respectively, 2 mm, 4 mm and 6 mm above the most coronal points of the gingival margin of the anterior anchorage teeth). Posteriorly, the relationship is reversed. Here, the width increase is greater in the early group patients at all measurement levels compared with the late group (highly significant *p* = 0.001, between the most coronal points of the gingiva of the 6-year molars as well as 2 mm above).

#### ***Ratio of width anterior to posterior (a/p, sagittal plane; dental, gingival-alveolar, skeletal-basal) (Table ******3******)***

**Table 3:** Ratio of anterior to posterior width (sagittal plane). Ratio a/p of the width on the dental, gingival-alveolar and skeletal-basal plane. ΔT2-T1 (Diff.) was determined from the ratio of the differences of the respective widths in the anterior and posterior area between the times T1 and T2. $\frac{\mathrm{Width a T}2-\mathrm{Width a T}1}{\mathrm{Width p T}2-\mathrm{Width p T}1}$. ΔT2-T1 (Diff.) < 1 indicates a greater increase in the posterior region, ΔT2-T1 (Diff.) = 1 shows an equal change anteriorly and posteriorly, ΔT2-T1 (Diff.) > 1 indicates a greater increase in the anterior region. *M* Mean, *SD* standard deviation, *a* anterior (1st deciduous or premolar), *p* posterior (1st molar), *GM* gingival margin

	**All patients**					**PG 1**					**PG 2**					**PG 1 vs. PG 2**
	T1	T2	ΔT2-T1	ΔT2-T1 (Diff.)		T1	T2	ΔT2-T1	ΔT2-T1 (Diff.)		T1	T2	ΔT2-T1	ΔT2-T1 (Diff.)		
	M ± SD	M ± SD	M ± SD	M ± SD	*p*(intra)	M ± SD	M ± SD	M ± SD	M ± SD	*p*(intra)	M ± SD	M ± SD	M ± SD	M ± SD	*p*(intra)	*p*(inter)
Ratio a/p dental	0.77 ± 0.04	0.79 ± 0.05	0.02 ± 0.04	1.26 ± 4.23	< 0.001	0.76 ± 0.03	0.77 ± 0.04	0.02 ± 0.03	0.70 ± 1.70	< 0.001	0.78 ± 0.04	0.81 ± 0.05	0.03 ± 0.04	1.78 ± 5.62	< 0.001	0.020
Ratio a/p GM gingival-alveolar	0.77 ± 0.05	0.80 ± 0.06	0.03 ± 0.05	1.54 ± 1.83	< 0.001	0.77 ± 0.05	0.79 ± 0.05	0.01 ± 0.04	0.97 ± 0.45	0.017	0.77 ± 0.06	0.82 ± 0.07	0.05 ± 0.05	2.11 ± 2.42	0.017	< 0.001
Ratio a/p -6 mm skeletal-basal	0.64 ± 0.13	0.69 ± 0.13	0.05 ± 0.13	0.84 ± 3.24	< 0.001	0.69 ± 0.11	0.72 ± 0.13	0.03 ± 0.11	0.57 ± 1.86	0.052	0.59 ± 0.12	0.65 ± 0.11	0.06 ± 0.08	1.12 ± 4.19	< 0.001	0.062

The ratio a/p is the quotient of the anterior to posterior differences in width between T1 and T2; it was determined at three exemplary levels. In the early group, there is a significantly greater increase in width in the posterior area than anteriorly at all three levels (*p* < 0.001; *p* = 0.017; *p* = 0.052). This indicates a slightly inverted V-shaped, delta-shaped widening of the palate. The opposite is true for the patients in the late group. Here, the increase at all levels is significantly greater anteriorly than posteriorly (*p* < 0.001; *p* = 0.017; *p* < 0.001), and thus, the suture opening occurs V-shaped. Between groups, the differences are significant at the dental level (*p* = 0.020) and highly significant at the gingival-alveolar level (*p* < 0.001).

#### ***Height (frontal plane) (Table ******4******)***

**Table 4:** Height (frontal plane). Heights (in mm) of the palate. In the anterior region, median (Raphe median line) and 5 mm right and left paramedian were measured; in the posterior region, median (Raphe median line) and 5 mm and 10 mm right and left paramedian were measured. *M* Mean, *SD* standard deviation, *RML* raphe median line, *ri* right (1st quadrant), *le* left (2nd quadrant), *ant.* anterior (1st deciduous or premolar), *post.* posterior (1st molar)

	**All patients**				**PG 1**				**PG 2**				**PG 1 vs. PG 2**
	T1	T2	ΔT2-T1		T1	T2	ΔT2-T1		T1	T2	ΔT2-T1		
	M ± SD	M ± SD	M ± SD	*p*(intra)	M ± SD	M ± SD	M ± SD	*p*(intra)	M ± SD	M ± SD	M ± SD	*p*(intra)	*p*(inter)
RML ant	10.32 ± 1.74	10.95 ± 1.89	0.63 ± 1.37	< 0.001	10.28 ± 1.67	10.74 ± 2.08	0.46 ± 1.23	< 0.001	10.36 ± 1.81	11.16 ± 1.67	0.81 ± 1.48	< 0.001	0.964
RML ant. 5 mm ri	8.08 ± 2.04	9.05 ± 2.25	0.97 ± 1.72	< 0.001	8.11 ± 1.96	8.93 ± 2.42	0.81 ± 1.67	< 0.001	8.05 ± 2.13	9.18 ± 2.07	1.13 ± 1.76	< 0.001	0.493
RML ant. 5 mm le	8.10 ± 2.06	8.98 ± 2.11	0.88 ± 1.53	< 0.001	7.95 ± 2.02	8.74 ± 2.17	0.79 ± 1.51	< 0.001	8.25 ± 2.11	9.23 ± 2.04	0.97 ± 1.55	< 0.001	0.277
RML post	13.11 ± 2.38	13.55 ± 2.62	0.44 ± 0.96	< 0.001	12.11 ± 2.43	12.55 ± 2.56	0.44 ± 1.00	< 0.001	14.11 ± 1.88	14.54 ± 2.31	0.43 ± 0.93	< 0.001	0.862
RML post. 5 mm ri	11.66 ± 2.35	12.14 ± 2.51	0.48 ± 1.17	< 0.001	10.62 ± 2.33	11.18 ± 2.41	0.56 ± 1.32	< 0.001	12.69 ± 1.88	13.10 ± 2.24	0.41 ± 1.01	< 0.001	0.263
RML post. 10 mm ri	6.27 ± 1.85	6.94 ± 2.19	0.67 ± 1.46	< 0.001	5.53 ± 1.41	6.05 ± 1.68	0.52 ± 1.26	< 0.001	7.02 ± 1.95	7.83 ± 2.30	0.82 ± 1.63	< 0.001	0.178
RML post. 5 mm le	11.94 ± 2.36	12.51 ± 2.66	0.57 ± 1.34	< 0.001	10.88 ± 2.30	11.47 ± 2.64	0.60 ± 1.36	< 0.001	13.00 ± 1.90	13.55 ± 2.26	0.55 ± 1.33	< 0.001	0.337
RML post. 10 mm le	6.79 ± 2.26	7.55 ± 2.56	0.76 ± 2.01	< 0.001	6.00 ± 1.85	6.55 ± 2.06	0.55 ± 1.97	< 0.001	7.58 ± 2.37	8.55 ± 2.64	0.97 ± 2.05	< 0.001	0.520

The height increases highly significant at each measurement point both anteriorly and posteriorly in both groups (*p* < 0.001). It is remarkable that the increases to the right and left laterally of the median palatine raphe are greater in absolute terms than at the raphe itself. There are no significant differences in the height changes between groups. In the sagittal, anterior–posterior comparison, the increase in height in the anterior region is more pronounced in the late group than in the posterior region. In the early group, however, the anterior and posterior height increases are almost equal.

### Angular measurement

#### ***Modified palatal index according to Kim ***et al***. ***[24]*** (Table ******5******)***

**Table 5:** Angle measurements (modified palatal index according to Kim et al. [24]). Angle in degrees (°) in the anterior and posterior region. According to the modified palatal index according to Kim et al. [24], a flat palate is present with an angle < 30°, a normal palate with an angle between 30 and 45° and a steep palate with an angle > 45°. *M* Mean, *SD* standard deviation, *ant.* anterior (1st premolar or deciduous molar), *post.* posterior (1st molar)

	**All patients**				**PG 1**				**PG 2**				**PG 1 vs. PG 2**
	T1	T2	ΔT2-T1		T1	T2	ΔT2-T1		T1	T2	ΔT2-T1		
	M ± SD	M ± SD	M ± SD	*p*(intra)	M ± SD	M ± SD	M ± SD	*p*(intra)	M/SD	M ± SD	M ± SD	*p*(intra)	*p*(inter)
**ant**	46.07 ± 5.01	42.90 ± 4.97	− 3.18 ± 4.47	< 0.001	46.60 ± 4.11	43.13 ± 4.82	− 3.47 ± 4.36	< 0.001	45.55 ± 5.76	42.67 ± 5.14	− 2.88 ± 4.59	< 0.001	0.483
**post**	38.05 ± 5.96	36.51 ± 6.39	− 1.54 ± 3.87	< 0.001	35.62 ± 5.75	33.10 ± 5.07	− 2.52 ± 4.07	< 0.001	40.48 ± 5.16	39.93 ± 5.74	− 0.56 ± 3.42	0.225	0.006

In the early group, the angle decreases highly significant (*p* < 0.001) in the anterior and posterior region, with these angle changes being almost equal in magnitude. In contrast to that, the angle decreases highly significant only in the anterior region in the late group (*p* < 0.001). Comparing the groups directly reveals a highly significant decrease of the angle in the posterior region in the early group than in the late group (*p* = 0.006).

## Discussion

### Transverse, frontal and sagittal changes

In the present clinical study, morphological changes of the palate were evaluated three-dimensionally after rapid maxillary expansion. The treatment effects of rapid maxillary expansion are largely determined by the therapeutic point of force application to the rotational centres of the maxilla. According to various studies, these centres are located dorsally in the area of the median palatine suture and close to the frontomaxillary sutures [26–31].

Many studies focus on changes within the transverse plane, especially on those of the median palatine suture. Both Bazargani et al. [10] and Liu et al. [32] found primarily no consensus in their systematic reviews as to whether RME treatment leads to triangular, i.e., greater anterior opening, or to a parallel widening of the median palatine suture. In the individual studies examined, however, measurement methods and recording techniques vary greatly. The results of the present clinical study in conjunction with the case studies suggest that the opening of the median palatine suture depends on the patient age at treatment onset: it opens approximately parallel in the early group and triangularly in case of a later therapeutic intervention. A parallel suture opening is also described in the studies by Christie et al. [33] and Podesser et al. [23] for patients with a chronological age of 10 years or less. Habersack et al. [34] also described age-dependent differences for the therapeutic effects of RPE treatment on median palatal sutures using CT data from two comparable cases: a 10-year-old patient with mixed dentition showed a parallel opening, whereas the 16-year-old patient with permanent dentition experienced a greater opening in the anterior region than in the posterior region and thus a triangular expansion.

A possible connection between patient age and opening mode of the median palatine suture seems obvious. Several authors attribute the decreasing expansion in the region of the first permanent molars with advancing age to the onset of ossification of the median palatal suture at this particular level [35–38]. It is undisputed that the median palatine suture is subject to age-related changes [39]. With increasing age, the degree of ossification increases and progresses from posterior to anterior, whereby the onset and progress of obliteration are subject to strong inter- and intraindividual variations [38, 40]. Wehrbein and Yildizhan [41] as well as Knaup et al. [37] were able to demonstrate in studies on human palates that even in advanced age only minor ossifications of the median palatine suture were present and that the mean sutural width also decreased only slightly compared to younger individuals. They concluded that an increased resistance to transverse expansion in adult patients was probably due to other factors, such as pronounced sutural interdigitations or increased bone rigidity. In a micro-CT analysis of 28 palatal specimens from humans aged 14–71 years, the only age-related factor determined was bone density (BV/TV [%]) in the sagittal plane among the other investigated parameters obliteration index in the frontal plane, suture length, linear sutural distance, interdigitation index in the horizontal plane and bone density. Thus, the morphology of the suture does not seem to have a limiting factor on the mode of opening but rather the bone density of the suturally adjacent maxillary bone [40] and the increasing rigidity of the pterygomaxillary pillars [42].

The triangular pyramidal maxillary expansion in the frontal plane has corresponding effects from the nasal cavity to the alveolar processes [43, 44] and includes orthopaedic and orthodontic components. In the present study, a triangular expansion of the maxilla between the dental plane, the gingival-alveolar plane and the skeletal basal plane is seen in the frontal plane in both the anterior and posterior regions in all patients (see Table 2). This “bending up of alveolar processes” has also been described in CT studies by Podesser et al. [23] and Weissheimer et al. [45] at least for the posterior region.

In the sagittal plane, the late group shows a greater height increase anterior than posterior, while in the early group, it is almost uniform. Although palatal growth is mainly genetically determined, other factors such as adjacent anatomical structures, growth- or therapy-related changes in the position of the teeth or the position of the tongue also play a role [46, 47]. In a Korean longitudinal study on digitised models, Yang et al. [48] documented the growth of the palate in untreated and non-treated subjects between the ages of six and 14. Both palate height—median and right and left paramedian—and width increase more in the posterior region than in the anterior region. Even though ethnic reasons make the comparison with the participants of this study difficult due to the Asian origin of the subjects, and despite differences in measurement points and distances, it is particularly remarkable that the palatal height increase after RPE treatment is different in both treatment groups of the present study. Five millimetres and 10 mm to the right and left of the median raphe, the lateral increase is always greater than at the median raphe itself in absolute terms. In addition, the height increase in the early group is the same anteriorly and posteriorly. In the late group, this is more pronounced in the anterior region than in the posterior region and thus even contrary to the results of the longitudinal growth study by Yang et al. [48].

The modified palatal index according to Kim et al. [24] documents highly significant changes in the shape of the palate in the anterior and posterior regions with early intervention. Patients in the late group show a highly significant flattening of the palatal morphology only anteriorly. These results are consistent with those in the transverse, frontal and sagittal planes.

### Possible relationship between changes in palatal morphology and degrees of obliteration of maxillary sutures

The therapeutical forces and moments generated through activation of the Hyrax screw act both upon the maxilla and on deeper cranial structures [18], especially the palatine bones and the pterygoid processes of the sphenoid bone. The rising tensions are initially concentrated on the anterior palate and then proceed dorsally along the median palatine suture and via the palatine bone to the sphenoid bone, the zygomatic processes and the medial orbital walls [14]. A possible reason for the triangular expansion found in adolescent patients may be that the anatomical proximity of the maxilla to the pterygoid processes of the sphenoid bone presents a rising resistance to the opening of the suture in the posterior region with increasing age [45, 49].

Holberg [42] used the finite element method (FME) to show that these stresses and deformations are only moderate in the juvenile sphenoid bone, whereas in adults, due to the decreasing elasticity of bony structures, the lateral bending up of both pterygoid processes in the area of the maxillary canal (foramen rotundum ossis sphenoidalis), the inferior maxillary foramen (foramen ovale ossis sphenoidalis) and the superior orbital fissure can cause considerable stresses, which could lead to microfractures with injury to nervous and vascular structures.

The interaction of the various centres of rotation is the cause of the therapeutically induced changes in the height and shape of the palate.

Age-dependent and in favour of the early intervention is the parallel and thus more even opening of the median palatine suture. Post-therapeutic functional stabilisation of the dilation through the establishment of a physiological swallowing pattern and harmonisation of the tongue rest position is thus more likely to be guaranteed with a uniform opening and is essential for long-term stability. In the case of a more V-shaped dilatation after late treatment, an increased caudal tongue rest position and thus an increased risk of recurrence due to a lack of functional stabilisation is to be expected [50].

## Limitations of the study

The results of the present retrospective study represent summation effects of natural growth and therapeutic effects. To determine the net treatment effects, the natural growth would have to be subtracted from each measured parameter. However, comparative data of untreated patients with the same initial findings (transverse maxillary arch deficiency) and the corresponding measurement distances over a corresponding period of time are neither available from a separate comparison group nor in historical growth studies to the necessary extent. It should also be borne in mind that natural growth is likely to be small during the average treatment period when compared to the extent of the therapeutic effects. In addition, the RPE appliances were in situ for retention for approximately the same length of time (approx. 6 months on average) in both patient groups. Out of a larger group, the same number of patients was divided equally between two groups according to their dentition stage, and it was ensured that the number of Hyrax screw activations was exactly identical. This was the only way to compare and discuss differential therapeutic effects between two treatment groups with different dentition and ossification stage.

The three-dimensional analysis of the plaster models implies different bony changes in dependence of the patients’ age. Further clinical studies with radiologically documentation of the changes must be conducted in order to confirm the anticipated skeletal reactions in this study.

## Conclusions

The present study comprehensively investigated the age-dependent three-dimensional treatment effects of rapid maxillary expansion on the morphology of the maxillary palate on virtual casts for the first time. If a parallel expansion of the maxilla is desired, a RME should be performed during the early mixed dentition.

## References

[1] Angell EC (1860). Treatment of irregularities of the permanent adult teeth. Dent Cosmos.

[2] Korbmacher H, Huck L, Merkle T, Kahl-Nieke B (2005). Clinical profile of rapid maxillary expansion – outcome of a national inquiry. J Orofac Orthop.

[3] Grabowski R, Hinz R, Stahl de Castrillon F (2009). Das kieferorthopädische Risikokind: Gebissentwicklung und Funktionsstörungen - KFO-Prävention und Frühbehandlung.

[4] Biederman W (1973). Rapid correction of class III malocclusion by midpalatal expansion. Am J Orthod.

[5] Haas AJ (1961). Rapid expansion of the maxillary dental arch and nasal cavity by opening the midpalatal suture. Angle Orthod.

[6] Lee KJ, Park YC, Park JY, Hwang WS (2010). Miniscrew-assis-ted nonsurgical palatal expansion before orthognathic surgery for a patient with severe mandibular prognathism. Am J Orthod Dentofacial Orthop.

[7] Ludwig B, Glasl B, Zorkun B, Wilmes B, Kinzinger G, Lisson J (2009). Forcierte Gaumennahterweiterung mit skelettalem Kraftansatz: Die Hybrid-GNE. Kieferorthop.

[8] Harzer W, Schneider M, Gedrange T (2004). Rapid maxillary expansion with palatal anchorage of the hyrax expansion screw–pilot study with case presentation. J Orofac Orthop.

[9] Winsauer H, Vlachojannis J, Winsauer C, Ludwig B, Walter A (2013). A bone-borne appliance for rapid maxillary expansion. J Clin Orthod.

[10] Bazargani F, Feldmann I, Bondemark L (2013). Three-dimensional analysis of effects of rapid maxillary expansion on facial sutures and bones: a systematic review. Angle Orthod.

[11] Ghoneima A, Abdel-Fattah E, Hartsfield J, El-Bedwehi A, Kamel A, Kula K (2011). Effects of rapid maxillary expansion on the cranial and circummaxillary sutures. Am J Orthod Dentofacial Orthop.

[12] Jain V, Shyagali TR, Kambalyal P, Rajpara Y, Doshi J (2017). Comparison and evaluation of stresses generated by rapid maxillary expansion and the implant-supported rapid maxillary expansion on the craniofacial structures using finite element method of stress analysis. Prog Orthod.

[13] Priyadarshini J, Mahesh CM, Chandrashekar BS, Sundara A, Arun AV, Reddy VP (2017). Stress and displacement patterns in the craniofacial skeleton with rapid maxillary expansion - a finite element method study. Prog Orthod.

[14] Chaconas SJ, Caputo AA (1982). Observation of orthopedic force distribution produced by maxillary orthodontic appliances. Am J Orthod Dentofacial Orthop.

[15] Gardner GE, Kronman JH (1971). Cranioskeletal displacements caused by rapid palatal expansion in the rhesus monkey. Am J Orthod Dentofacial Orthop.

[16] Leonardi R, Sicurezza E, Cutrera A, Barbato E (2011). Early post-treatment changes of circumaxillary sutures in young patients treated with rapid maxillary expansion. Angle Orthod.

[17] Shetty V, Caridad JM, Caputo AA, Chaconas SJ (1994). Biomechanical rationale for surgical-orthodontic expansion of the adult maxilla. J Oral Maxillofac Surg.

[18] Starnbach H, Bayne D, Cleall J, Subtelny JD (1966). Facioskeletal and dental changes resulting from rapid maxillary expansion. Angle Orthod.

[19] Garib DG, Henriques JFC, Janson G, Freitas MR, Coelho RA (2005). Rapid maxillary expansion - tooth tissueborne versus tooth-borne expanders: a computed tomography evaluation of dentoskeletal effects. Angle Orthod.

[20] Timms DJ (1980). A study of basal movement with rapid maxillary expansion. Am J Orthod.

[21] Baratieri C, Alves MJR, Gomes de Souza MM, Araujo M, Maia LC (2011). Does rapid maxillary expansion have long-term effects on airway dimensions and breathing?. Am J Orthod Dentofacial Orthop.

[22] Maspero C, Galbiati G, Del Rosso E, Farronato M, Giannini L (2019). RME: effects on the nasal septum. A CBCT evaluation. Eur J Paediatr Dent.

[23] Podesser B, Williams S, Crismani AG, Bantleon HP (2007). Evaluation of the effects of rapid maxillary expansion in growing children using computer tomography scanning: a pilot study. Eur J Orthod.

[24] Kim SJ, Donovan DM, Blanchard SB, Kowolik JE, Eckert GJ (2008). The relationship between acute otitis media and the anatomic form of the hard palate. Pediatr Dent.

[25] Dahlberg G (1940). Statistical methods for medical and biological students.

[26] Baldini A, Nota A, Santariello C, Caruso S, Assi V, Ballanti F, Gatto R, Cozza P (2018). Sagittal dentoskeletal modifications associated with different activation protocols of rapid maxillary expansion. Eur J Paediatr Dent.

[27] Bucci R, D'Antò V, Rongo R, Valletta R, Martina R, Michelotti A (2016). Dental and skeletal effects of palatal expansion techniques: a systematic review of the current evidence from systematic reviews and meta-analyses. J Oral Rehabil.

[28] Haas AJ (1965). The treatment of maxillary deficiency by opening the midpalatal suture. Angle Orthod.

[29] Tausche E, Deeb W, Hansen L, Hietschold V, Harzer W, Schneider M (2009). CT analysis of nasal volume changes after surgically-assisted rapid maxillary expansion. J Orofac Orthop.

[30] Wertz R (1970). Skeletal and dental changes accompanying rapid midpalatal suture opening. Am J Orthod.

[31] Wertz R, Dreskin M (1977). Midpalatal suture opening: a normative study. Am J Orthod.

[32] Liu S, Xu T, Zou W (2015). Effects of rapid maxillary expansion on the midpalatal suture: a systematic review. Eur J Orthod.

[33] Christie KF, Boucher N, Chung CH (2010). Effects of bonded rapid palatal expansion on the transverse dimensions of the maxilla: a cone-beam computed tomography study. Am J Orthod Dentofacial Orthop.

[34] Habersack K, Karoglan A, Sommer B, Benner KU (2007). High-resolution multislice computerized tomography with multiplanar and 3-dimensional reformation imaging in rapid palatal expansion. Am J Orthod Dentofacial Orthop.

[35] Cross DL, McDonald JP (2000). Effect of rapid maxillary expansion on skeletal, dental, and nasal structures: a postero-anterior cephalometric study. Eur J Orthod.

[36] Kajan ZD, Nasab NK, Eghrari N (2018). Quantitative evaluation of midpalatal suture opening and its relation with zygomaticomaxillary suture status in patients aged 7–25 years using cone beam computed tomography images: in an Iranian population. Contemp Clin Dent.

[37] Knaup B, Yildizhan F, Wehrbein H (2004). Age-related changes in the midpalatal suture. A histomorphometric study. J Orofac Orthop.

[38] Persson M, Thilander B (1977). Palatal suture closure in man from 15 to 35 years of age. Am J Orthod Dentofacial Orthop.

[39] Melsen B (1975). Palatal growth studied on human autopsy material. Am J Orthod.

[40] Korbmacher H, Schilling A, Püschel K, Amling M, Kahl-Nieke B (2007). Age-dependent three-dimensional microcomputed tomography analysis of the human midpalatal suture. J Orofac Orthop.

[41] Wehrbein H, Yildizhan F (2001). The mid-palatal suture in young adults. A radiological-histological investigation. Eur J Orthod.

[42] Holberg C (2005). Effects of rapid maxillary expansion on the cranial base – an FEM-Analysis. J Orofac Orthop.

[43] Bell RA (1982). A review of maxillary expansion in relation to rate of expansion and patient’s age. Am J Orthod.

[44] Majourau A, Nanda R (1994). Biomechanical basis of vertical dimension control during rapid palatal expansion therapy. Am J Orthod Dentofacial Orthop.

[45] Weissheimer A, de Menezes LM, Mezomo M, Dias DM, de Lima EM, Rizzatto SM (2011). Immediate effects of rapid maxillary expansion with Haas-type and hyrax-type expanders: a randomized clinical trial. Angle Orthod.

[46] Heiser W, Niederwanger A, Bancher B, Bittermann G, Neunteufel N, Kulmer S (2004). Three-dimensional dental arch and palatal form changes after extraction and nonextraction treatment. Part 3. Transversal and sagittal palatal form. Am J Orthod Dentofacial Orthop.

[47] Jimenez P, Martinez-Insua A, Franco-Vazquez J, Otero-Cepeda XL, Santana U (2012). Maxillary changes and occlusal traits in crania with artificial fronto-occipital deformation. Am J Phys Anthropol.

[48] Yang ST, Kim HK, Lim YS, Chang MS, Lee SP, Park YS (2013). A three dimensional observation of palatal vault growth in children using mixed effect analysis: a 9 year longitudinal study. Eur J Orthod.

[49] Gautam P, Valiathan A, Adhikari R (2007). Stress and displacement patterns in the craniofacial skeleton with rapid maxillary expansion: a finite element method study. Am J Orthod Dentofacial Orthop.

[50] Volk J, Kadivec M, Mušič MM, Ovsenik M (2010). Three-dimensional ultrasound diagnostics of tongue posture in children with unilateral posterior crossbite. Am J Orthod Dentofacial Orthop.

